# Acute Effect of Exposure to Extreme Heat (100 ± 3 °C) on Lower Limb Maximal Resistance Strength

**DOI:** 10.3390/ijerph191710934

**Published:** 2022-09-01

**Authors:** Ignacio Bartolomé, Víctor Toro-Román, Jesús Siquier-Coll, Diego Muñoz, María C. Robles-Gil, Marcos Maynar-Mariño

**Affiliations:** 1 Faculty of Health Sciences, University Isabel I, 09003 Burgos, Spain; 2 School of Sport Sciences, University of Extremadura, Avenida de la Universidad s/n, 10003 Cáceres, Spain; 3 SER Research Group, Center of Higher Education Alberta Giménez (Affiliated to Comillas Pontifical University), 07011 Palma de Mallorca, Spain

**Keywords:** hyperthermia, acute exposure, muscular strength, performance, repetition maximum

## Abstract

The aim of this study was to evaluate the acute effect of a single dry sauna bath lasting twelve minutes on the indirect determination of the one maximum repetition (1RM) leg press among trained and untrained participants. Thirty young men participated in the study, a trained group (TG; *n* = 15; age: 20.97 ± 0.44 years) and an untrained group (UG; *n* = 15; age: 21.03 ± 0.11 years). Subjects in the TG had performed resistance training for at least two years before the beginning of the experiment. All participants performed two indirect tests of their one maximum repetition leg press on two different days, with a rest period of three weeks between tests. Additionally, anthropometric, body composition, blood pressure, body temperature, and rated perceived exertion were evaluated. On the second testing day, all of the participants took a dry sauna bath lasting 12 min immediately before performing the leg press test. In the second evaluation (pre-heating in the sauna), the UG experienced increases in absolute RM (178.48 ± 56.66 to 217.60 ± 59.18 kg; *p* < 0.05; R = 0.798), relative RM (2.65 ± 0.61 to 3.24 ± 0.58 kg·g body mass^−1^; *p* < 0.05; R = 0.798), and muscular RM (5.64 ± 1.20 to 6.77 ± 1.14 kg·kg muscle mass^−1^; *p* < 0.05; R = 0.797). The TG also increased their values on the second day in absolute RM (284.96 ± 62.41 to 314.92 ± 1.04 kg; *p* < 0.01; R = 0.886), in relative RM (3.61 ± 0.88 to 3.99 ± 1.85 kg*kg body mass^−1^; *p* < 0.01; R = 0.886), and muscular RM (7.83 ± 1.69 to 8.69 ± 1.85 kg·kg muscle mass^−1^; *p* < 0.01; R = 0.854). A passive, extreme-heat sauna bath lasting 12 min taken immediately before a relative maximum repetition test seems to provoke clear positive responses for the development of strength.

## 1. Introduction

Sauna baths have been used all over the world for hundreds of years for different purposes, such as the promotion of health, as well as social, hygienic, and spiritual practices. It has been proven that sauna bathing induces several beneficial physiological and molecular responses that are linked to the cardiovascular, immunological, nervous, and endocrine systems [1], as well as to muscle force production [2].

In recent reviews, it has been found that heat stress can be induced using different types of devices and/or techniques. In this sense, exposure to heat and acclimatization to heat can be achieved through the use of thermal cameras, infrared devices, hot water baths, or saunas [1]. In the case of saunas, thermal stress can be configured by combining temperature and relative humidity (RH). Depending on the configuration of RH, dry sauna baths can be differentiated from steam baths or wet saunas [3].

Although the specific effects on the human body may differ from one technique to another, the main responses are always linked to the stresses suffered by the thermoregulatory, cardiovascular, circulatory, and perspiratory systems [4]. In this sense, when a human being heats up, heat begins to accumulate in the human body, and the temperature of all tissues and cells tends to increase [5]. In order to prevent overheating, heat stroke, or even death, the humoral, nervous, and sensory systems stimulate thermoregulatory responses in order to remove heat from the body through sweating, conduction, and evaporation [6].

In terms of acute responses to heat, these mechanisms induce increases in heart rate, sweat rate, respiratory rate, and volume, as well as a change in blood circulation, and decreases in blood pressure and body water content [6].

However, if heat exposure is planned and repeated over several days, and if hydration is controlled, the human body begins to acclimatize to heat [5]. The immediate consequence of acclimatization is an increase in general resistance to heat; all systems, tissues, and cells become less affected by environmental heat [6].

Additionally, the effects of heating muscles or the entire body on physical performance have been widely studied in the last few decades. It has been demonstrated that muscle strength and contractile properties are correlated with muscle tissue temperature [7]. Furthermore, physical benefits have been associated with passive warming in the context of muscle strength development [8].

Similarly, the endocrine effects of sauna bathing have been widely investigated in the last few decades in several European countries, reporting a beneficial effect of heat in the secretion of hormones linked to the “fight” response to stress [9,10].

Additionally, in the last few years, muscle strength has been linked to heat treatment, especially because of the anabolic responses that are induced by muscle heating [11]. For this reason, heat acclimation is beginning to be considered as a promoter of muscle anabolism in the context of muscle hypertrophy [1], with the majority of the publications that are linked to this topic.

However, in the context of athletic performance, most of the investigations are linked to the detrimental role of hyperthermia on physical performance, as well as to the effects of acclimating subjects in order to avoid heat-induced declines in physical performance, especially among endurance athletes [12].

Regarding the development of neuromuscular strength, it has been recently observed that low-intensity strength training in extremely hot conditions (100 ± 3 °C) for three weeks induced several responses were linked to the production of force, concluding a positive effect of heat exposure upon the development of maximal isometric strength [13]. However, the acute effect of heat exposure on maximal strength is still unclear, and evidence exists that supports a positive heat-induced response.

Finally, it has been demonstrated that trained humans can respond and adapt to heat in a significantly different way than untrained ones [14]. The reasons for these differences are linked to the adaptive effects that training induces on the nervous, circulatory, endocrine, thermoregulatory, and neuromuscular systems [15]. In this sense, it has also been reported that trained humans tolerate heat to a greater degree than sedentary or untrained ones [16]. For this reason, and considering the current lack of knowledge about the acute effects of extreme heat in humans, it is interesting to evaluate the effects of extreme heat in two clearly differentiated types of populations: trained and untrained participants.

Thus, the aim of this study was to evaluate the acute effect of a single exposure to extreme heat (100 ± 3 °C) on the indirect determination of maximal strength in a leg press exercise among both trained and untrained participants.

## 2. Materials and Methods

### 2.1. Ethical Guidelines

The study protocol was reviewed and approved by the Biomedical Ethics Committee of the University of Extremadura (Spain) (Code: 32/2020). The study was designed and carried out following the ethical guidelines of the Helsinki Declaration for Human Research, updated in the World Medical Assembly of Fortaleza (2013).

### 2.2. Health Security Protocol

Before the beginning of the study, every participant was examined by a physician. The medical exam consisted of a general medical aptitude exam, an electrocardiographic analysis using a digital electrocardiograph (Sanro, BTL-08 SD ECG, Pozuelo del Alarcón, Madrid, Spain), blood pressure screening using a digital tensiometer (Visomat; comfort 20/40, Külsheim, Germany), and an oxygen saturation test using an infrared pulse oximeter (Gima, Oxy-50, Gessate, Italy), during resting conditions and immediately after the performance of twenty burpees.

### 2.3. Participants

A total of 30 young men participated in this study. All of them were informed about the aims, characteristics, and risks of this study. All subjects participated voluntarily and gave their written informed consent before the beginning of the study. A numeric code was assigned to each participant in order to preserve their anonymity.

Additionally, all participants were required to fit the general inclusion criteria: to be a healthy man; to not be taking drugs, tobacco, or alcohol frequently; to be between 18 and 24 years old; to not be taking any nutritional supplements or pharmacological treatments; and to not have had any injury or illness in the previous 3 months.

In order to know the different effects of heat on trained and untrained subjects, the participants were deliberately recruited and assigned to two different groups according to their characteristics. The specific inclusion criteria for the participants in the trained group (TG: *n* = 15, age: 20.97 ± 0.44 years) were as follows: having performed resistance training for at least two years before the beginning the study, with a weekly frequency of 3–5 sessions. In contrast, participants in the untrained group (UG: *n* = 15, age: 21.03 ± 0.11 years) had never performed any kind of resistance or strength training protocol. The characteristics of both groups are presented in Table 1.

### 2.4. Experimental Protocol

**Figure 1:**
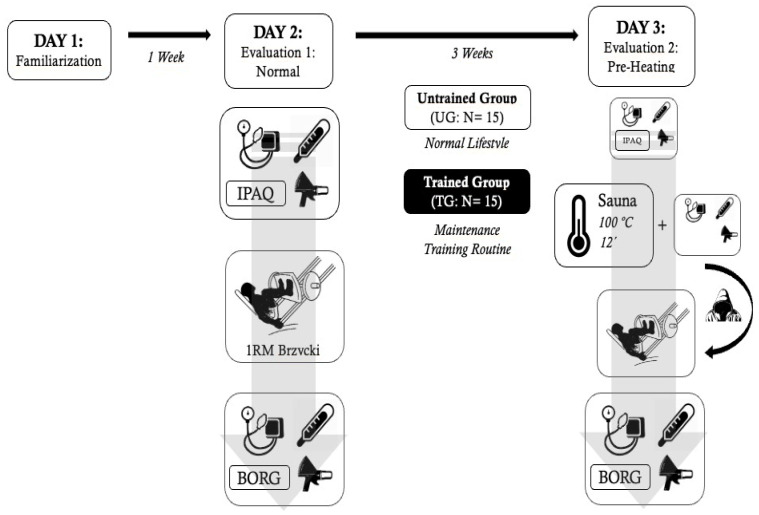
General study design.

The study protocol was performed on three different days. On the first day, every participant visited the laboratory and the fitness room at the Sport Sciences School in order to get acquainted with the equipment and facilities. On the second day, body composition and anthropometric characteristics, body temperature, blood pressure and heart rate, as well as indirect maximal strength were evaluated for each participant. Finally, on the third day, the same evaluations as on the second day were performed again but including a sauna bath session immediately before the strength test. All the testing procedures and techniques are described in the following sections. The entire study design is represented in Figure 1.

After the first evaluation (Day 2), the UG participants were instructed to continue their normal lifestyle until the second evaluation (Day 3). The TG counterparts were instructed to follow a maintenance training routine. They were allowed to maintain the exercises and the weekly program and schedule, but had to adapt their main training variables: volume, intensity, frequency, and density, in order to achieve a maintenance training stimulus. Training volume was reduced by 20%, individual intensities were set to 70% of their loads from the last week prior to the study, frequency was set to two days per week, and training density was reduced by 25%.

### 2.5. Anthropometric and Body Composition Evaluations

On each day, the height of each participant was evaluated using a wall-mounted stadiometer (Seca 220, Hamburg, Germany). Body weight was evaluated with a bioimpedance analyzer (Seca 769, Hamburg, Germany) in nude, barefoot conditions, and body mass index was calculated for each participant. At the same time, body composition and basal metabolism were evaluated with the bioimpedance analyzer, following the manufacturer’s guidelines.

Finally, six skinfolds (abdominal, supra-iliac, subscapular, tricipital, thigh, and calf) were evaluated using a manual plicometer (Holtain, Crymych, UK), and five body perimeters (arm, thigh, calf, waist, and hip) were measured using a calibrated measuring tape (Seca, Hamburg, Germany). The waist-to-hip ratio was calculated by dividing the waist perimeter by the hip perimeter. All anthropometric measurements were performed by the same operator, skilled in kinanthropometry techniques, and following the guidelines of the International Society of Kinanthropometry. Additionally, body composition calculations were performed using the formulas purposed by the International Society of Kinanthropometry [17].

Additionally, the body weights of all participants were assessed after the strength tests on both evaluation days, as well as after the sauna bath on the second evaluation day.

### 2.6. Physical Activity Evaluation

Physical activity was assessed using the International Physical Activity-Short Form Questionnaire (IPAQ-SF; Spanish version) [18]. The questionnaire was administered by email the week before the beginning of the study, and participants were previously advised about completion of the questionnaire.

### 2.7. Cardiovascular Evaluation

Systolic and diastolic blood pressure as well as heart rate were evaluated using a pulsometer (Polar, Vantage M, Kempele, Filand) and a digital arm tensiometer (Visomat; comfort 20/40, Külsheim, Germany). These cardiovascular evaluations were performed in resting conditions and after the strength test. On the second day, the cardiovascular evaluations were performed under resting conditions, after the sauna bath and after the strength test. All measurements were performed on the left arm.

### 2.8. Body Temperatures Evaluation

Body temperature was evaluated using an infrared thermometer [TAT 5000 “Exergen Temporal Scanner” (Watertown, MA, USA)]. The temperature was evaluated on the lateral wall inside the mouth, with the mouth completely closed; on the forehead; and at the mid-point of the external part of the dominant thigh. Body temperatures were evaluated on both study days. On day one, all temperatures were measured in resting conditions and immediately before the strength test. On day two, temperatures were evaluated identically, as well as just after the sauna bath. The skin was also dried with a tissue before applying the thermometer.

### 2.9. Strength Evaluation and Maximum Repetition Calculation

On both evaluation days, maximum strength was tested in the 45° leg press exercise (TELJU; Toledo; Spain). The evaluation consisted of the indirect calculation of the maximum repetition (*RM*), following the guidelines of Brzycki [19]. The *RM* was calculated by using the original Brzycki formula, shown as follows:

$$Predicted\;RM=\frac{Weight\;Lifted}{1.0278-0.278\times N}$$

where *N* is the number of repetitions completed.

In order to avoid the possible influence of body position and technique, the configuration of the leg press machine was saved for each participant and reproduced identically on the second day of evaluation. The performance of each repetition was assessed by an evaluator who was skilled in resistance exercises.

Additionally, the warm up as well as the loading protocols used were the same on both days; participants performed an initial light aerobic warm up of 5 min on the static bike, followed by 3 short dynamic stretches of the hamstrings. Then, the 1RM protocol began with one set of 15 repetitions without load on the leg press. Then, the guidelines of Brzycki were applied in order to set the approximation series as well as the final one.

Once the participant reached voluntary failure, the load was noted as well as the maximum number of valid repetitions. Calculations of 1RM were performed using the Brzycki formula. Then the test was stopped, and body temperature and blood pressure were immediately evaluated with the participant still in the leg press apparatus. Finally, maximum relative strength was calculated by dividing absolute 1 RM (in kg) by body (relative RM: kg/kg body mass) and muscle (muscular RM: kg/kg muscle mass) masses.

### 2.10. Rating of Perceived Exertion

In both evaluations, and immediately after the test, all participants gave their rating of perceived exertion (RPE) using the Borg Scale [20]. Subjects indicated a number on a 10-point scale from 0 (very very very light) to 10 (very very very hard).

### 2.11. Sauna Bathing

On the second day, after the initial evaluation of anthropometric characteristics, blood pressure and body temperature, all participants took a passive dry sauna bath. A Finnish sauna (Harvia, C105S Logix Combi Control; 3–15 W; Muurame, Finland) was used for the sauna baths. Once the sauna reached 100 ± 3 °C, the participants went individually into the sauna with a towel. The RH did not change, maintaining the normal environmental values of the region (45–50%).

The participants had to be seated for 12 min under hot conditions. After this time, body temperature, blood pressure, and body weight were evaluated. Then the participants changed into sports clothing and went to the gym on the floor above.

Water and beverage intake was limited on day 3. No participant was allowed to drink anything during the 90 min before the sauna bath, as well as during the sauna session and strength tests.

### 2.12. Moving from the Sauna to the Gym

In order to avoid cooling of the body and the loss of the immediate effect of heat, each participant was required to go to the gym in the shortest possible time, and with his head covered with the towel that was previously heated in the sauna. The time taken to move from the sauna to the gym was recorded, being on average 35.12 ± 6.05 s, with the maximum time taken being 40.05 ± 1.17 s.

### 2.13. Statistical Analyses

IBM^®^ SPSS^®^ (Armonk, NY, USA) Statistics version 24 for Macintosh^®^ was used to perform the statistical analyses. The analyses initially consisted of a normality test using the Shapiro–Wilk test and an outlier study using a box plot analysis. The Mann–Whitney U test was applied to find differences between both study groups on each evaluation day. Then, the Wilcoxon test for paired samples was used to calculate differences between evaluation days in each group separately. Finally, the effect size was calculated using Cohen’s r for non-parametric data [21].

## 3. Results

Table 1 shows the general characteristics of both study groups. The table shows all anthropometric, body composition, metabolic, and fitness variables on both study days. The TG showed higher values in body weight (*p* < 0.05), BMI (*p* < 0.05), muscle and lean (*p* < 0.05) weights, and body water content (*p* < 0.05) on both evaluations. Additionally, this group presented greater values in weekly training volume (*p* < 0.01) and body perimeters: arm (*p* < 0.001), thigh (*p* < 0.01), and calf (*p* < 0.05), in comparison to the UG. No differences were found between study days in any group.

Table 2 presents the different physiological responses to the strength test and sauna bath for each group on both evaluation days. The UG did not experience any change after the RM test. However, on the second day, a decrease in body weight (*p* < 0.05) and an increase in HR (*p* < 0.05) and all body temperatures (*p* < 0.05) were observed among the untrained participants. After the RM test on the second evaluation, the UG experienced a decline (*p* < 0.05) in mouth and forehead temperatures. At this moment, HR was significantly higher (*p* < 0.05) than pre-sauna values.

Additionally, comparing post-RM values of the UG between days showed that the diastolic blood pressure was lower (*p* < 0.05) on the second day, while HR and mouth temperature were higher (*p* < 0.05).

Regarding the TG on the first evaluation day, only an increase in HR (*p* < 0.05) was observed after the RM test; all the other variables were unaltered. In contrast, on the second day after the sauna bath, a decrease in body weight (*p* < 0.01), and an increase in both HR (*p* < 0.01) and all body temperatures (*p* < 0.01) were observed. After the RM test on the second evaluation, the TG experienced a reduction in all body temperatures (*p* < 0.01), while the final values of HR (*p* < 0.01), mouth (*p* < 0.01), and thigh (*p* < 0.05) temperatures were higher in comparison to the respective pre-sauna ones. The final diastolic blood pressure values obtained on the second evaluation were lower (*p* < 0.05) than the final ones on the first evaluation, while HR and mouth temperature were higher (*p* < 0.05).

Finally, if both study groups are compared, it can be observed that the TG showed greater body weight (*p* < 0.05) during the study; the only physiological variable that differed between groups was mouth temperature, being lower among the TG participants after the second evaluation of RM.

Table 3 shows the results obtained in the strength evaluations. The table shows the values of each variable on both study days, as well as the differences between days expressed in absolute values (differences) as well as in percentages (% change). Additional size effect calculations are shown (R). All of these parameters are expressed for each group.

Regarding the responses of the UG on evaluation 2 (pre-heating), increases in the load (*p* < 0.05; R = 0.797), absolute RM (*p* < 0.05; 0.798), relative RM (*p* < 0.05; R = 797), and muscular RM (*p* < 0.05; 0.797) were observed, although the number of repetitions and the RPE were unaltered.

The TG also experienced on evaluation 2 increases in load (*p* < 0.01; R = 0.888), absolute RM (*p* < 0.01, R = 0.886), relative RM (*p* < 0.01; R = 0.886), and muscular RM (*p* < 0.01; R = 0.854). Additionally, this group also experienced an increase in RPE (*p* < 0.05; R = 0.630) on the second day.

If both study groups are compared, the TG presented higher values in load on both days (*p* < 0.01), with the % change in this parameter being lower (*p* < 0.05) among TG participants. The absolute RM was also higher among the TG on evaluation 1 (*p* < 0.001) and evaluation 2 (*p* < 0.05). The relative RM was higher only on evaluation 1 (*p* < 0.01), while muscular RM was higher on both evaluation 1 (*p* < 0.01) and evaluation 2 (*p* < 0.05) as well.

## 4. Discussion

This paper aimed to evaluate the effect of a single acute exposure to extreme heat (100 °C) on the development of strength and several physiological variables in both trained and untrained participants.

Interestingly, in the 1970s, 1980s, and 1990s, several studies were conducted that observed a positive effect of passive heating on the development of strength and maximal anaerobic power [22,23,24]. In the last few years, this field has been scarcely studied and the use of extreme heat has only recently been implemented for the first time in the context of strength development [13].

In this research, a clear effect of a sauna bath has been shown in strength and physiological parameters for both trained and untrained subjects. As far as it can be found in the literature, no previous study has been conducted that used the acute effect of extreme passive heating on the development of maximal resistance strength; however, several studies can be considered in the discussion of the obtained results.

In the first place, to carry out this study, two groups with clearly differentiated physical characteristics and training levels were deliberately recruited. It should be noted that physical training is, as has been universally accepted, a highly influential variable in maximum strength tests performance. In this sense, the significantly higher values obtained by the TG in all the strength variables show, firstly, a clear effect of resistance training and, secondly, that these groups constitute valid participants to obtain consistent data on the effects of extreme heat in both types of participants.

In this investigation, both study groups significantly increased their loads when a sauna bath was implemented before the RM test. The strength gain was +39.1 ± 34.5 kg (+26.7 ± 33.5%; *p* < 0.05; R = 0.798) in the UG, and +30.0 ± 21.0 kg (+11.0 ± 7.2%; *p* < 0.01; R = 0.886) among the TG participants, which manifest a positive effect of acute extreme heat among trained and untrained humans. In relation to these findings, Hedley et al. evaluated the effect of a passive dry sauna bath on the development of maximal strength on both upper and lower limbs [25]. In their study, they observed a decrease of 4% in the leg press exercise, while the values of the bench press remained stable in comparison to the normal values. Although these authors examined the effect of heat upon maximal strength, the thermal load was significantly different from the load that was used in the present study. They used a longer thermal period (30 min) than that used in this experiment (12 min), along with a considerably lower dry temperature (70 °C in comparison to 100 °C). This is manifested in the thermoregulatory load, because the average weight loss by sauna bath in the present study was 0.3 kg (UG: 66.43 ± 8.13 vs. 66.25 ± 8.12 kg; TG: 80.47 ± 12.19 vs. 80.15 ± 12.10 kg), a weight loss that was similar to the one observed by Hedley et al. [25]. This possibly implies greater neuro-thermoregulatory stimulation, which may be directly related to force production; considering the greater volume of heat exposure in the study of Hedley et al., this may have induced neuromuscular fatigue in the subjects of their study.

As the heat volume was considerably lower in the present study, another explanation should be considered: heat stress involves great hypothalamic stimulation in thermoregulatory responses, but also beta-adrenergic and sympathetic activity, which has been demonstrated during sauna baths [26]. It has been observed that passive sauna bathing at 80 °C can induce dramatic increases in several hormones linked to beta-adrenergic and sympathetic activities (adrenaline, noradrenaline, cortisol), as well as increases in other stress hormones such as adrenocorticotropic hormone, prolactin, or even growth hormone [9,10]. Catecholamines have been shown to be involved in responses to tolerate stress and pain [27], as well as in the ability of the neuromuscular system to produce force [28]. It is worth mentioning here that in both groups, the RPE increased in the second evaluation compared to the first. Among the TG subjects, this increase reached statistical significance (*p* < 0.05), and may be related to the role of these stress-induced hormones that are highly responsive to heat [29].

Interestingly, it has also been reported that heat stress may affect the CNS as well as its different tissues [30]. When nerves are heated, the neurotransmission speed augments, increasing neural drive and stimulation of motor plates [31,32,33]. The underlying mechanism is not completely understood yet, but it seems clear that it can be attributed to the specific nature of the nerve tissues, as well as to the actions of several heat-induced proteins [34,35]. It has been observed that when nerve tissue temperature increases, P2X and TPRV1 proteins modulate its activity, generating an analgesic response which may be highly beneficial in resistance exercises. The P2X proteins are membrane receptors that are located in several tissues, such as sensorial nerves innervating muscle tissues [36]. These proteins are activated in cases of inflammatory or tissular damage, stimulating sensorial nerves and generating a pain response which inhibits muscle contraction [37]. It has been demonstrated that increases in muscle or nerve temperatures may inhibit P2X activity, inducing an analgesic response [38].

In addition, TPRV1 proteins are cell membrane protein channels linked to nociception. These proteins act by modulating the sensitivity of the cells to several cations, as well as generating and sending pain signals to the CNS [39]. TPRV1 activity is modulated by several factors, with tissue temperature playing a critical role [40]. It has been reported that heat and increases in sensorial nerve temperatures augment the activity of these channels, modulating nerve membrane potentials that can affect the responses of the circulatory, respiratory, and neuromuscular systems [41,42], as well as the subjective perception of effort and fatigue development [43]. In parallel, it has been demonstrated that the positive heat-induced response in neurotransmission is notably higher in type IIB fibers [35,44]. However, these molecular responses are still not entirely clear in humans, and although they may have contributed to increased force production, further research in this field is required in order to draw firm conclusions.

It is interesting that after the pre-heating in the sauna, body temperature increased significantly in both study groups (UG: 39.05 ± 2.11 °C, *p* < 0.05; TG: 39.36 ± 1.65 °C, *p* < 0.01), and that the TG participants increased their RPE (*p* < 0.05; R = 0.630) after the pre-heating RM test in comparison to the RM test without pre-heating. First of all, it should be mentioned that post-sauna body temperatures exceeded 38 °C in both groups, which has been considered in the literature as hyperthermia [5].

Considering that resistance strength tests and exercises induce dramatic increases in mechanical tension [45], metabolic stress [46], and muscular damage [47], and that these kinds of motor situations are limited by sensorial and effort perception systems [45], the responses of both above-mentioned families of proteins contribute to the obtained results.

Additionally, in the last few years, several cellular and molecular mechanisms linked to strength development have been proven to be stimulated by heat stress. One important finding in this field is the response of the heat shock proteins (HSP) in the motor plates, where these proteins increase the resistance of the plate to general and heat stressors, and facilitate the release and uptake of neurotransmitters [33,48,49].

Linked to this response is the behavior of catecholamines, which are highly sensitive to heat stress [10,26]. Catecholamines increase the beta-adrenergic activity of muscles, accelerating metabolism and increasing the rates of synthesis and acetylcholine release in motor plates [50]. Considering that all body temperatures increased in both untrained (*p* < 0.05) and trained (*p* < 0.01) participants, the roles of these stress hormones should be considered.

In the interpretation of the strength results, it should also be considered that in human muscles, heat acts as an enzymatic accelerator [51], increasing the activity of numerous enzyme families, such as myosin ATPase [52] or creatine kinase [53]; these enzymes are highly limiting in maximal strength development [45,54]. It has also been demonstrated that by passively heating muscle, the neuromuscular capacity to generate force can be increased by 5% for each 1 °C increase, in comparison to thermoneutral tissues in both resistance [22] and explosive [23] muscle contractions. Considering that neutral temperatures in human tissues range between 36 and 37.5 °C [55], and that in the present study the temperatures of the skin of the legs reached 39 °C in both groups of subjects, it is likely that these metabolic mechanisms contributed to the observed increases in strength after stimulation in the sauna.

It has also been reported that maximal anaerobic power is enhanced by passive heating. Davies and Young found that local pre-heating increased peak power in maximal efforts of 10 s [23], as well as in maximum-intensity sprints of 20 s, where average power also increased [56].

Dehydration has also been linked to heat and its relationship to force production. In explosive strength and power exercises, such as jumping, an improvement in performance related to controlled dehydration induced by heat stress has been reported [57]. In this sense, it has been observed that a loss in body fluids of less than 4% of body weight (BW) can be helpful in jumping exercises [57]. However, dehydration is also related to loss of body and/or muscle water content, as well as various electrolytes. It has been reported that modulation of these parameters can negatively affect muscle performance, especially when dehydration is severe. It is widely accepted that severe losses in electrolytes and/or body water content can reduce physical performance [58].

In the present study, the reduction in body weight (BW) induced by sauna baths, although significant, was significantly less than 4% of BW (UG: 66.4 ± 8.1 to 66.2 ± 8.1 kg; TG: 80.5 ± 12.2 to 80.2 ± 12.2 kg).

For all these reasons, it should be mentioned that although an increase in performance has been observed in the present investigation, it has not been possible to analyze the content of body water or the levels of electrolytes in blood or sweat. Taking into account the relevance of electrolytes and hydration status in physical performance and heat stress, this fact may constitute a limitation of this study. In future research, it will be of great interest to evaluate body fluid and electrolyte status after sauna bathing, as well as the specific relationships of these variables with the production of force.

Regarding structural mechanisms, strength development is partially dependent on muscle mass, fiber structural protein content, and the cross-bridging rate [45]. Heat can also modulate muscle viscosity, lubrication, and structural properties [59], thereby modifying the spatial conformations of actin filaments and increasing the binding capacities of contractile proteins [60]; this accelerates the rate of cross-binding [30]. It has to be mentioned that heat stress increases the elastic properties of skeletal muscles, augmenting their tolerance to mechanical loads by means of a modulation in collagen structures [61]. All of these heat-induced responses are closely linked to resistance exercises and force production, and may explain the obtained results as well as provide an explanation as to why trained subjects experienced greater effects of heat upon relative and muscular RM.

The obtained data showed positive cardiovascular responses after exposure to high temperatures (lower blood pressure). It is well known that maximal strength exercises or high loaded resistance exercises can generate a hypertensive response that makes this type of work inadvisable in some populations [15]. It should be highlighted that the sauna bath previous to the RM test led to a decline in post-test diastolic blood pressure in both trained and untrained participants (*p* < 0.05), in comparison to the respective values of the post-test that did not involve a prior sauna bath. This response can be explained by the activity of nitric oxide (NO); its vasodilator and antihypertensive effects have been widely reported [62], as well as its great sensitivity to heat stress [63].

Finally, the relationship of the training level with the acute effect of heat should be considered. It has already been previously mentioned that two clearly differentiated groups were recruited in this study. This fact is manifested in the values of the maximum strength test in the first evaluation, with the TG obtaining higher values in the Load (*p* < 0.01), absolute RM (*p* < 0.001), relative RM (*p* < 0.01) and muscular RM (*p* < 0.01) than UG. In relation to the training effect, it has to be highlighted that although after the sauna bath the TG again obtained higher values than the UG, the differences were statistically much smaller both in the load (*p* < 0.01) and in all RM values (*p* < 0.05).

Unfortunately, these differences cannot yet be exactly explained. However, it is known that physical training increases body temperature [62] and this could partly explain these results.

Due to these systematic elevations in body temperature as a consequence of the weekly training sessions, the TG participants could have developed higher heat resistance [14] than those corresponding to the UG. This could have caused a greater sauna-induced stimulation among the UG participants, due to not being familiar with the thermal stress induced by exercise. In any case, the data obtained in this regard open an interesting line of research that should be deepened in subsequent studies.

In summary, the results obtained in this study have shown several positive effects of acute extreme heat on some variables that are of great relevance in the context of force production and evaluation. For this reason, these results may be of interest to athletes in strength and power modalities, as well as to practitioners of counter-resistance training. However, more research is required on this topic in order to understand the potential benefits associated with the implementation of sauna baths for improvements in the performance of sports and in competitions involving strength and power.

Additionally, findings in this field are scarce and novel, and although this paper manifests a clear acute response to heat in the development of maximal resistance strength, the results should be considered with caution. More studies are necessary to obtain clear conclusions on the application of acute heat stress before strength and resistance tests.

## 5. Conclusions

It can be concluded that acute extreme heat (100 °C) stimulation in a dry sauna previous to maximal strength testing led to an increase in indirect maximal absolute as well as relative strength, in both trained and untrained subjects.

This increase in strength was accompanied by a decrease in diastolic blood pressure in all participants, as well as by an increase in the subjective perception of effort among trained participants. These results show a clear acute effect of heat on strength development; however, further research is required.

## Figures and Tables

**Table 1:** Body composition, anthropometric, and fitness characteristics of participants in the study.

	Evaluation 1: Normothermia		Evaluation 2: Pre-Heating	
	Untrained Group (*n* = 15)	Trained Group (*n* = 15)	Untrained Group (*n* = 15)	Trained Group (*n* = 15)
Weight (kg)	66.45 ± 8.03	80.26 ± 12.44 ^+^	66.56 ± 8.22	80.58 ± 12.34 ^+^
Height (m)	1.74 ± 0.05	1.76 ± 0.09	1.74 ± 0.05	1.76 ± 0.09
BMI	21.82 ± 2.46	24.97 ± 2.21 ^+^	21.83 ± 2.55	25.05 ± 2.17 ^+^
BM (Kcal/day)	1706 ± 111	1925 ± 210 ^+^	1707 ± 121	1930 ± 208 ^+^
Training (hours/week)	4.56 ± 0.92	9.17 ± 1.02 ^++^	4.72 ± 0.71	10.22 ± 0.43 ^++^
Perimeter-Arm (cm)	28.97 ± 1.73	33.70 ± 2.79 ^+++^	28.72 ± 1.75	33.73 ± 3.03 ^+++^
Perimeter-Tight (cm)	51.65 ± 3.16	57.57 ± 4.70 ^++^	51.12 ± 4.17	57.58 ± 3.99 ^++^
Perimeter-Calf (cm)	36.12 ± 2.62	38.36 ± 2.27 ^+^	35.82 ± 2.53	38.79 ± 1.93 ^+^
Perimeter-Waist (cm)	75.93 ± 3.96	79.04 ± 6.23	75.81 ± 4.42	79.66 ± 5.43
Perimeter-Hip (cm)	83.42 ± 7.51	85.20 ± 7.31	81.66 ± 6.78	84.73 ± 6.96
Waist-to-hip index	0.91 ± 0.06	0.92 ± 0.02	0.93 ± 0.03	0.92 ± 0.02
Fold-Abdominal (cm)	19.45 ± 8.62	18.13 ± 8.44	19.71 ± 7.56	18.41 ± 9.37
Fold-Suprailiac (cm)	16.43 ± 7.35	17.20 ± 7.76	16.01 ± 8.10	18.95 ± 7.93
Fold-Subscapular (cm)	13.31 ± 5.38	13.37 ± 4.96	13.42 ± 6.34	13.51 ± 3.77
Fold-Tricipital (cm)	11.80 ± 4.78	12.16 ± 4.34	11.56 ± 4.30	13.75 ± 5.75
Fold-Tight(cm)	17.62 ± 7.64	2086 ± 7.83	17.35 ± 6.62	19.55 ± 6.29
Fold-Twin (cm)	9.67 ± 4.33	11.16 ± 4.39	9.15 ± 4.46	11.94 ± 5.64
Fat weight (kg)	8.16 ± 2.61	10.45 ± 4.25	8.08 ± 2.51	10.71 ± 4.34
Fat weight (%)	12.20 ± 3.48	12.27 ± 3.62	12.10 ± 3.28	12.96 ± 3.51
Muscle weight (kg)	31.54 ± 4.69	38.21 ± 4.25 ^+^	31.52 ± 5.03	38.00 ± 4.57 ^+^
Muscle weight (%)	51.28 ± 2.65	51.14 ± 3.06	50.29 ± 2.80	50.66 ± 3.42
Lean weight (kg)	58.36 ± 7.21	69.81 ± 8.74 ^+^	58.46 ± 7.22	69.86 ± 8.92 ^+^
Lean body Mass (%)	87.02 ± 4.08	83.31 ± 3.64	86.21 ± 4.50	83.55 ± 4.84
Bone weight (kg)	11.04 ± 0.92	12.25 ± 1.89	11.03 ± 0.74	12.44 ± 1.84
Bone weight (%)	16.73 ± 1.45	15.30 ± 1.19	16.70 ± 1.41	15.47 ± 0.96
Body water (kg)	42.16 ± 3.74	48.77 ± 6.44 ^+^	41.96 ± 3.66	48.98 ± 5.54 ^+^
Body Water (%)	63.68 ± 2.97	60.97 ± 2.68	63.32 ± 3.27	61.17 ± 3.55

BMI: body mass index. BM: basal metabolism. Mann–Whitney U Test: differences between groups in each day: ^+^ *p* < 0.05; ^++^ *p* < 0.01; ^+++^ *p* < 0.001.

**Table 2:** Physiological effects of strength evaluation and sauna exposure.

	Evaluation 1: Normothermia		Evaluation 2: Pre-Heating		
	Pre-RM	Post-RM	Pre-Sauna	Post-Sauna- Pre-RM	Post-RM
	Untrained Group (*n* = 15)				
Weight (kg)	66.4 ± 8.0	66.4 ± 7.5	66.4 ± 8.1	66.2 ± 8.1 *	66.6 ± 8.8
SBP (mmHg)	125 ± 17	136 ± 21	120 ± 5	123 ± 12	122 ± 12
DBP (mmHg)	73 ± 7	78 ± 10	73 ± 12	73 ± 6	68 ± 8 °
Heart Rate (bpm)	82 ± 17	93 ± 19	73 ± 12	125 ± 20 *	123 ± 25 ^°
Temp-Mouth (°C)	36.6 ± 0.5	37.1 ± 0.7	36.9 ± 0.4	39.0 ± 0.8 *	38.2 ± 0.9 *°
Temp-Forehead (°C)	36.7 ± 0.5	36.5 ± 0.4	36.8 ± 0.4	39.6 ± 2.1 *	36.8 ± 1.4 *
Temp-Tight (°C)	35.7 ± 0.5	35.6 ± 0.5	34.9 ± 1.8	39.1 ± 2.1 *	36.5 ± 1.5
	Trained group (*n* = 15)				
Weight (kg)	80.3 ± 12.4 ^+^	80.4 ± 10.1 ^+^	80.5 ± 12.2 ^+^	80.2 ± 12.2 ^+^**	80.1 ± 12.4 ^+^
SBP (mmHg)	128 ± 12	128 ± 9	122 ± 9	119 ± 11	123 ± 9
DBP (mmHg)	75 ± 6	74 ± 9	72 ± 14	76 ± 18	71 ± 9 °
Heart Rate (bpm)	73 ± 10	84 ± 16 *	66 ± 7	113 ± 19 **	106 ± 17 ^^°
Temp-Mouth (°C)	36.4 ± 0.6	36.7 ± 0.4	36.6 ± 0.5	38.5 ± 0.9 **	37.3 ± 0.44 ^+^**^^°
Temp-Forehead (°C)	36.7 ± 0.4	36.5 ± 0.5	36.6 ± 0.5	39.8 ± 1.4 **	36.3 ± 0.3 **
Temp-Tight (°C)	34.4 ± 1.9 ^+^	35.4 ± 0.6	34.3 ± 2.3	39.4 ± 1.7 **	35.7 ± 0.3 **^

SBP: systolic blood pressure. DBP: diastolic blood pressure. Temp: temperature. Mann–Whitney U test: differences between groups in each day: ^+^ *p* < 0.05; Wilcoxon test: differences for each study group in different evaluations; differences between consecutive evaluations of days 1 and 2: * *p* < 0.05; ** *p* < 0.01; differences between pre-sauna and post-RM of day 2: ^ *p* < 0.05; ^^ *p* < 0.01; differences between post-RM of days 1 and 2: ° *p* < 0.05.

**Table 3:** Results of strength evaluation.

	Evaluation 1: Normothermia	Evaluation 2: Pre-Heating	*Differences.*	% Change	R
Untrained Group (*n* = 15)					
Load (kg)	161.9 ± 49.7	198.8 ± 50.7 *	+36.8 ± 27.6	26.9 ± 28.9	0.797
Repetitions	4.3 ± 0.9	4.0 ± 1.2	−0.3 ± 1.4	−2.3 ± 35.1	0.169
RM (kg)	178.5 ± 56.7	217.6 ± 59.2 *	+39.1 ± 34.5	26.7 ± 33.5	0.798
Relative RM	2.7 ± 0.6	3.2 ± 0.6 *	+0.6 ± 0.6	26.4 ± 33.0	0.798
Muscular RM	5.6 ± 1.2	6.8 ± 1.1 *	+1.1 ± 1.1	23.8 ± 32.3	0.797
RPE	8.0 ± 0.9	8.4 ± 0.9	+0.4 ± 0.7	5.1 ± 9.8	0.421
Trained group (*n* = 15)					
Load (kg)	257.054.9 ^++^	282.0 ± 59.8 ^++^**	+25.0 ± 6.5	9.9 ± 6.2 ^+^	0.888
Repetitions	4.5 ± 0.7	4.8 ± 0.4	+0.3 ± 0.7	8.8 ± 17.0	0.424
RM (kg)	285.0 ± 62.4 ^+++^	314.9 ± 1.0 ^+^**	+30.0 ± 21.0	11.0 ± 7.2	0.886
Relative RM	3.6 ± 0.9 ^++^	4.00 ± 1.9 ^+^**	+0.4 ± 0.3	10.5 ± 7.2	0.886
Muscular RM	7.8 ± 1.7 ^++^	8.7 ± 1.9 ^+^**	+0.9 ± 0.5	11.4 ± 6.6	0.854
RPE	7.5 ± 0.7	8.3 ± 0.8 *	+0.80 ± 1.0	11.5 ± 14.7	0.630

RM: maximum repetition. Mann–Whitney U test: differences between groups in each day: ^+^ *p* < 0.05; ^++^ *p* < 0.01; ^+++^ *p* < 0.001. Wilcoxon test: differences between days 1 and 2 for each study group: * *p* < 0.05; ** *p* < 0.01. R: effect size between days for each group separately.

## Data Availability

Not applicable.
